# E-Cigarettes for Immediate Smoking Substitution in Women Diagnosed with Cervical Dysplasia and Associated Disorders

**DOI:** 10.3390/ijerph13030288

**Published:** 2016-03-04

**Authors:** Shirley A. James, Ellen M. Meier, Theodore L. Wagener, Katherine M. Smith, Barbara R. Neas, Laura A. Beebe

**Affiliations:** 1 Department of Biostatistics and Epidemiology, College of Public Health, University of Oklahoma Health Sciences Center, Oklahoma City, OK 73104, USA; barbara-neas@ouhsc.edu (B.R.N.); laura-beebe@ouhsc.edu (L.A.B.); 2 Department of Pediatrics, University of Oklahoma Health Sciences Center, Oklahoma City, OK 73104, USA; emeier@ostatemail.okstate.edu (E.M.M.); theodore-wagener@ouhsc.edu (T.L.W.); 3 Oklahoma University Physicians, University of Oklahoma Health Sciences Center, Oklahoma City, OK 73104, USA; katie-smith@ouhsc.edu

**Keywords:** smoking cessation, cervical dysplasia, electronic cigarette, EC, electronic nicotine delivery device, vaping

## Abstract

The aim of this study was to determine if 31 women with cervical dysplasia and associated conditions exacerbated by smoking would be successful substituting cigarettes with their choice of either nicotine replacement therapy (NRT) or electronic cigarettes (EC). Women received motivational interviewing and tried both NRT and ECs, choosing one method to use during a six-week intervention period. Daily cigarette consumption was measured at baseline, six, and 12 weeks, with differences analyzed by the Wilcoxon signed-rank test. Study analysis consisted only of women choosing to use ECs (29/31), as only two chose NRT. At the 12-week follow-up, the seven day point prevalence abstinence from smoking was 28.6%, and the median number of cigarettes smoked daily decreased from 18.5 to 5.5 (*p* < 0.0001). The median number of e-cigarette cartridges used dropped from 21 at the six-week follow-up to 12.5 at the 12-week follow-up. After initiating EC use, women at risk for cervical cancer were able to either quit smoking or reduce the number of cigarettes smoked per day. Although a controlled trial with a larger sample size is needed to confirm these initial results, this study suggests that using ECs during quit attempts may reduce cigarette consumption.

## 1. Introduction

Despite being a preventable disease, cervical cancer is the 4th most common cancer in women worldwide [1]. The precursor to cervical cancer is cervical dysplasia [2,3]. The human papilloma virus (HPV) is a necessary, though not sufficient, cause of cervical dysplasia [2,3,4,5]. While the presence of HPV is independent of smoking status, smoking plays a critical role in its progression to invasive cervical disease [3,4,5,6]. Since smoking is a known risk factor for cervical cancer, smoking cessation is a key intervention for all women diagnosed with HPV or cervical dysplasia. Smoking cessation is also vital after cervical cancer diagnosis, as continued smoking significantly increases the risk of all-cause and cancer-specific mortality [7]. No studies of smoking cessation in this population have been reported.

To date, the US Food and Drug Administration (FDA) has approved several products, including nicotine replacement therapy (NRT) and prescription drugs that do not contain nicotine, for treating tobacco use and dependence [8]. Despite the availability of several approved products for smoking cessation, the ratio of former smokers to ever smokers in the US has remained relatively unchanged from 48.7% in 1998 to 51.1% in 2008 [9]. Although not currently approved by the FDA for the treatment of tobacco dependence, electronic cigarettes (EC) have recently become popular, both for smoking reduction and nicotine substitution purposes. ECs can provide a coping mechanism for smokers who miss the smoking cues regular cigarettes provide, including hand to mouth simulation, as well as “drawing” the vapor into the lungs [10]. Though the long-term benefits and risks have yet to be determined, electronic cigarettes are generally accepted by public health professionals to pose less risk than cigarettes since they do not combust [11]. Two randomized controlled trials have suggested ECs may contribute to smoking cessation, or at least be as effective as the nicotine patch [12,13]. In two longitudinal studies smokers not interested in quitting either reduced their combustible cigarette use up to 44% or quit smoking entirely [14,15]. Given the causal link between the progression of dysplasia and smoking [3,4,5,6], it is important to identify effective ways to eliminate or substitute the use of combustible tobacco among women with cervical dysplasia and associated diagnoses. 

The purpose of this study was to examine the impact of nicotine replacement therapy and electronic cigarettes on smoking reduction and substitution in women at-risk or diagnosed with cervical dysplasia. 

## 2. Experimental Section

Participants: Subjects included 31 patients from the Stephenson Cancer Center Dysplasia Clinics who tested positive for HPV, or had recently been diagnosed with cervical dysplasia, cervical cancer, or lower genital tract dysplasia. The physician in charge of the clinic determined medical eligibility and referred women to the study. Study personnel then screened each subject, explaining the opportunity to try two unnamed smoking reduction products and to choose one for a 6-week smoking reduction study. Inclusion criteria were females who were smokers, aged 18–65 years. Smokers were defined as those who in a typical week smoked an average of 3 cigarettes a day or more and had done so for at least the last year. Exclusion criteria included patients unwilling to commit to a 6-week intervention; current diagnoses of or treatment for other cancer; presence of any known stroke, heart disease, or high blood pressure not well controlled with medication; pregnancy; lactating or planning pregnancy in the next 6 months; or current use of ECs or vaping systems. The study was approved by the Institutional Review Board (IRB) at the University of Oklahoma Health Sciences Center (#2506). 

**Figure 1:**
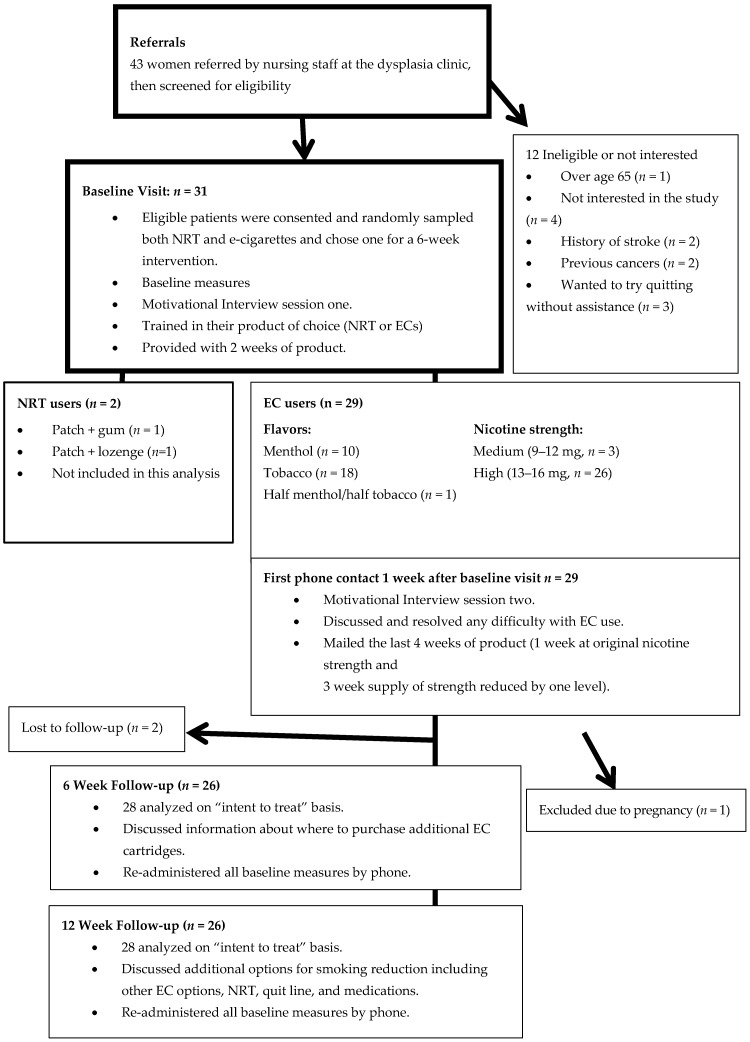
Flow of the study.

Procedures: After an initial screen for eligibility, women were consented and completed baseline assessments. At the initial visit, women tried ECs, nicotine gum, and nicotine lozenges, using random assignment to determine order. They did not try the nicotine patch, although many had used it in their past. During product presentation, study personnel explained how the product should be used to maximize effectiveness, with no attempt to bias the participant’s choice. She also explained the potential benefit of each product, possible side effects, and how to avoid them. After trying both kinds of products, women chose the method they preferred to use for a 6-week intervention period. We anticipated that women would choose either method somewhat equally; however, 29 of the 31 women (93.5%) chose ECs. Thus, while 31 women participated, the study population for this analysis includes only women who chose ECs (Figure 1). 

Each woman received smoking cessation motivational interviewing (MI) at her initial visit and during a follow-up phone call one week later. During the MI sessions the dangers of smoking were explained, along with an explanation of how cigarette smoke can interact with cervical dysplasia to cause a progression in the number and size of the lesions. Subjects also discussed their readiness to quit smoking, as well as potential barriers to smoking reduction/cessation, and how to avoid them. MI sessions were tailored to the use of ECs for smoking reduction and substitution. 

In accordance with a previous study finding that the “Blu^®^” brand of EC was preferred by most subjects [15], women received a “Blu^®^” EC starter kit containing 2 batteries, chargers, and a 2-week supply of their choice of tobacco or menthol flavored nicotine cartridges according to manufacturers’ recommendations. The number of cartridges each woman received was determined by multiplying 1.5 times the number of packs she smoked per day [15]. After sampling the different nicotine strengths, women were allowed to select the strength they felt would be most helpful to them, choosing from high strength, containing between 13 and 16 mg of nicotine, medium strength containing between 9 and 12 mg of nicotine, or low strength containing 6–8 mg of nicotine. According to the manufacturer, each cartridge accommodates approximately 250 puffs [16]. After completion of the telephone MI session a week after enrollment, an additional 1-week supply of product at the original nicotine strength was mailed to the participant, along with an additional 3-week supply of cartridges with nicotine strength decreased by one level. The decrease in nicotine content was done to help women reduce their dependence on nicotine. At six and twelve weeks following enrollment, women were contacted via telephone and study personnel re-administered baseline measures. 

Participants received six weeks of intervention consisting of two MI sessions and a 6-week supply of EC cartridges, followed by 6-weeks of no intervention. During the no intervention period participants were free to use any EC cartridges they had not used previously from the study, or Blu^®^ cartridges that they purchased on their own, but were asked not to use other smoking cessation products or methods. Participants received $20 cash after completion of the baseline assessment and were mailed $20 gift cards after completing both the 6- and 12-week follow-ups by phone. 

Measures: To assess smoking behaviors women were asked, “In the last week, on average, how many cigarettes did you smoke each day?” If the answer was none at follow-up, the women were asked the date they smoked their last cigarette, “even a puff.” During follow-up phone calls women were also asked how many EC cartridges they had used. EC use was recorded as the total number of cartridges each woman used during the previous 6-week period.

Additional measures included general demographics, baseline smoking behaviors, and the Fagerstrom Test for Cigarette Dependence (FTCD) [17,18]. The FTCD has been shown to be a reliable [17,18] measure of nicotine dependence with scores ranging from 0, indicating no dependence to 10, indicating a very high dependence on cigarettes. In order to measure participant’s motivation and confidence in their ability to quit smoking the Motivation and Confidence Rulers [19,20] and the Contemplation Ladder [21] were also used. The Motivation and Confidence to quit smoking scales both range from 0, indicating low levels, to 10, indicating high levels of either motivation or confidence. Measures of motivation have been independently associated with cessation (*p* < 0.001) [19,20]. The Contemplation Ladder is also scaled from 0 to 10 with higher scores reflecting a higher level of contemplation of quitting smoking. Finally a depression screen, the 9-item Patient Health Questionnaire (PHQ-9) was administered, with scores ranging from 0 to 27 [22]. Smoking cessation can exacerbate depression, thus women with a score of 10 or more on the PHQ-9 were referred to their doctor for intervention [23].

Statistical analysis: Summary descriptive statistics were calculated for each of the following assessments at baseline, 6-weeks, and 12-weeks: median daily cigarettes smoked, 7-day point prevalence abstinence, e-cigarette cartridges used during each measurement period, and scores on the FTCD, the PHQ-9, the Contemplation Ladder, and the Motivation and Confidence Rulers. To ascertain whether significant changes occurred, the nonparametric Wilcoxon signed-rank test was used to compare changes in medians from baseline to 6-week and baseline to 12-week periods [24]. At the 12-week follow-up only, the Wilcoxon signed-rank test was used to determine if changes in median cigarettes smoked and e-cigarette cartridges used were statistically significant in the smaller population of women who continued to both smoke cigarettes and use ECs at the end of the study. SAS 9.3 (SAS Institute, Cary, NC, USA) was used for all analyses with an alpha of 0.05 for statistical significance. Each subject was analyzed on intent to treat basis. Data missing due to loss to follow-up were treated as if there was no change from the baseline assessment. 

## 3. Results

Although 29 women chose the EC for the intervention period, one was excluded due to pregnancy at the one week follow-up. The remaining 28 women who chose the EC for substitution comprise the study population. Two of these 28 were lost to follow-up (Figure 1), both stating they did not have the time/energy to participate. Forty-six percent of participants had some level of college education, but none were college graduates, with 82% having a household annual income of $25,000 or lower. Twenty-nine percent of women in the study had no insurance, while 18% had private insurance (Table 1). 

Women scored a median of 9 out of 10 on the Motivation Ruler, 8 out of 10 on the Confidence Ruler, and 7 out of 10 on the Contemplation Ladder (Table 2). Women had a median initial score on the PHQ-9 of 4.5, indicating mild depression and a median initial score on the FTND of 5.0, indicating moderate dependence on nicotine (Table 2). 

Women smoked a median 18.5 cigarettes per day (Table 3) and the median number of years smoked was 20 (Table 1). 

None owned an e-cigarette or vaping system, although several had tried these devices previously. Nine women had tried to quit smoking in the past with the patch, one with gum, and seven with a combination of methods.

The seven day point prevalence abstinence from smoking combustible cigarettes was 14.3% (*n* = 4) and 28.6% (*n* = 8) at 6 and 12-week follow-up, respectively. Additionally, all subjects who were abstinent at 6-week remained abstinent at 12-weeks (Table 3). During the six-week intervention period, the median number of cigarettes smoked daily among the 28 women decreased from 18.5 cigarettes per day (cpd) to 6 cpd (*p* < 0.0001) and further dropped over the six-week follow-up period to 5.5 cpd (*p* < 0.0001 reduction from baseline) (Table 3). The median number of EC cartridges used among women continuing to use the EC dropped from 21 during the intervention period to 12.5 during the follow-up period (Table 3). A separate analysis of women who used both ECs and combustible cigarettes at the end of the study (*n* = 16) showed a reduction in median number of cigarettes smoked per day of 13 (*p* = 0.0005) with an accompanying decrease in EC cartridges used of 10.5 (*p* = 0.008) during the time from the beginning of the study to the 12-week follow-up. 

The median Fagerstrom score decreased from the initial value of 5.0, to 2.5 during intervention (*p* = 0.001), and 2.0 during follow-up (*p* < 0.0001), indicating a change from medium to low dependence on nicotine. The contemplation ladder changed from the baseline median of seven to the six-week median of eight (*p* = 0.04), which was retained at 12p weeks (*p* = 0.07).

## 4. Discussion

This study was the first to offer women at-risk or diagnosed with cervical cancer the opportunity to choose either NRT or ECs during a 6-week intervention period designed to reduce consumption of combustible cigarettes. The median number of daily cigarettes consumed by women in this study dropped from a baseline of 18.5 to 5.5. Although a larger controlled trial is needed to confirm our findings, this study suggests that ECs may have potential for inducing smoking reduction or cessation among women at-risk or diagnosed with cervical cancer. 

Although we anticipated that women would choose either NRT or ECs somewhat equally, the vast majority (93.5%) chose ECs following initial experimentation. Thus our results were restricted to the women who chose ECs. One explanation may be that most of the women in this study had tried different forms NRT in the past including the nicotine patch, lozenge, and gum, without success, making them prone to discouragement or burnout with NRT. An additional explanation may be that the women found ECs easy to use and appealing, both requirements for successful smoking cessation according to the Clinical Practice Guideline for Treating Tobacco Use and Dependence (2008) [25]. Many women had tried a friend’s EC or vaping device previous to the study and were curious about trying the device for themselves. Previous surveys of EC users indicate that consumers find ECs high in satisfaction and acceptability [26,27] and helpful for smoking reduction and/or cessation [26,27,28,29], with fewer perceived health risks than smoking [30]. In prior studies, consumers also noted that ECs lack the smell of regular cigarettes, but provide the valuable physical cues of hand to mouth simulation [30]. Finally, women in this study may have had been drawn to the EC because they found it to be a novel device that could potentially help them to reduce their intake of combustible cigarettes.

The seven day point prevalence for abstinence from cigarettes doubled from 14% at the 6-week follow-up to 29% at the 12-week follow-up. Median EC cartridge use dropped from 21 to 12.5. These results contradict EC studies in which EC use increases as cigarette smoking decreases. This result may be partially explained by the goal of most women in this study, which was to stop nicotine use altogether because of their diagnoses. Women were instructed to use ECs as a vehicle to do so, first substituting some or all of their cigarettes with ECs, and then trying to cut down on EC use as well. A total of eight women stopped smoking and four of these also quit using ECs. Twelve women became dual users of tobacco and ECs, substituting some of their cigarettes with EC cartridges. Our study found that both EC use and cigarette smoking were reduced, providing evidence that not all smokers who quit smoking with ECs continue to use ECs. 

Smoking substitution in this group of women persisted post intervention into follow-up when no study products were provided. Most women in this study (57%) continued to use ECs, providing evidence that the women found ECs acceptable as a nicotine replacement technique. Evidence for a decrease in cigarette dependence was provided by the women’s scores on the FTCD which were significantly decreased from a median of 5 at baseline to a median of 2.5 at 6 weeks and 2.0 at 12 weeks. 

This study contributes to and expands the evidence from other small uncontrolled studies of ECs for smoking reduction or cessation. Caponnetto and associates found sustained 50% reduction in cigarettes smoked per day in seven of 14 participants with schizophrenia with a median reduction from 30 to 15 cpd (*p* = 0.018) [31]. Pacifici and associates focused their eight-month pilot study on teaching study subjects who smoked to use ECs appropriately and at the end of the first, fourth, and eighth month found 73.5%, 50%, and 52.9% of participants respectively had switched to EC use only [32]. Polosa *et al.* surveyed daily cigarette consumption in 71 smokers making their first purchase at vape shops and at a 12-month follow-up found 40.8% had quit smoking and 25.4% had reduced by 50% or more [33]. 

Smoking cessation studies predict that smokers with low income levels, low education levels, poor medical insurance coverage, mild depression, and moderate levels of nicotine addiction will have more difficulty with smoking cessation than their counterparts [34]. Eighty-two percent of women in this study had a median yearly family income less than $25,000 dollars, which compares to the US level of poverty for a family of four of $24,250 [35]. Additionally, none had a college level education, and 29% had no medical insurance coverage of any kind. Despite having characteristics which predict poor smoking cessation outcomes, women using ECs in our study had positive outcomes. Possibly contributing to their success was that they were highly motivated, confident, and ready to quit [36], conceivably due to fear from their new diagnoses of a smoking-related medical condition. Boudreaux and associates found similar effects in their study of emergency room patients with diagnoses related to smoking [37]. They explored contributions of fear and perceived illness severity to intent to quit smoking. After adjusting for other covariates, event-related fear was significantly associated with intent to quit (*p* < 0.01, and perceived illness severity was correlated with event related fear (*p* < 0.001) but was not associated with intentions to quit (*p* = 0.32) [37].

Although contradictory evidence exists, one concern noted in the literature about EC use is that users will become more addicted to nicotine by becoming dual users [38,39]. While almost 43% of the women in this study remained dual users at the 12-week follow-up, both combustible cigarette and EC use did decrease significantly during the follow-up portion of the study. Additionally, the FTCD decrease in this study provides evidence that nicotine addiction was not enhanced by EC use in these women. These results are consistent with a study of vape store customers who, after initiation of ECs, reported decreases in their use of combustible cigarettes as well as decreases in nicotine concentrations in their e-liquid [40]. Another concern is that while studies associate the combustion from smoking with the progression of HPV to invasive cervical cancer, nicotine itself has been found in the cervical mucosa of women who smoke, but with unclear implications [5,6]. However, nicotine delivery in the form of NRT or the EC is safer than that of combustible cigarettes [11]. Moreover, studies demonstrate that over time many EC users are able to decrease their nicotine exposure [15]. 

This study has limitations that warrant discussion. First, this study comprised an uncontrolled small sample of convenience. In future studies, a randomized controlled trial with both a larger sample size and a longer follow-up period can help determine if ECs are effective for combustible cigarette replacement and abstinence. While another limitation of our study was a lack of biochemical verification of smoking status, high levels of both reliability (up to 97%) [41] and validity [42] (sensitivity of 100% and specificity of 95%) were found in studies comparing self-reported smoking status to expired air CO and urinary cotinine, respectively. 

At the point of discontent with ECs, women in our study may have been more successful if they had been allowed to try a different electronic nicotine delivery product. One example is a second generation vaping device that provides larger doses of nicotine more quickly, and satisfies nicotine craving more efficiently [40]. Using these devices with advice and technical support may have been an effective vehicle for these women [33]. The clinical practice guidelines [25] recommend that smokers try multiple smoking cessation techniques until they find one that they are confident will work for them. 

Despite its limitations, this study adds to the growing body of literature that suggests the EC may be a viable method for smoking reduction and cessation. It may hold particular promise for patients with cancer or other diagnoses in which smoking cessation is essential, including patients receiving radiation therapy and chemotherapy. 

## 5. Conclusions

Women with cervical dysplasia and associated diagnoses should be advised to stop smoking immediately to avoid proliferation and exacerbation of dysplasia lesions. Because smoking is addictive, this is a daunting task. In this study, 29 of 31 women chose to use ECs, providing evidence that ECs can be an acceptable substitute for smoking. EC use in this study was also proven to be effective in smoking substitution, as the median number of cigarettes smoked daily decreased from 18.5 to 6 (*p* < 0.0001), and further dropped over the six-week follow-up period to 5.5 (*p* < 0.0001 reduction from baseline). This study also provided evidence that nicotine addiction decreased with EC use. However, a substantial number of women were not successful in reducing or eliminating their smoking. Additional research is needed to elucidate the most effective methods of smoking cessation in high-risk populations such as those with precancerous diagnoses. 

## Figures and Tables

**Table 1:** Participant characteristics (*n* = 28).

Age-Categorical	Number	Percent
≤30 years	9	32.1%
31–50 years	12	43.0%
>50 years	7	25.0%
Number of years of smoking history	Number	Percent
<11 years	4	14.3%
11 to <20 years	8	28.7%
≥20 years	16	57.1%
Race	Number	Percent
White	20	71.4%
African American	4	14.3%
Native American	3	10.7%
Hispanic	1	3.6%
Annual Income	Number	Percent
<$25,000	23	82.1%
$25,001–$50,000	5	17.9%
>$50,000	0	0%
Insurance Coverage	Number	Percent
Medicaid only	11	39.3%
No insurance	8	28.6%
Private insurance	5	17.9%
Combination Medicare and Medicaid	4	14.3%
Education	Number	Percent
Less than High School	7	25.0%
High School graduate or GED only	8	28.6%
Some college (no degree)	13	46.4%
College degree	0	0%

**Table 2:** Initial, 6, and 12-week scores on standardized assessments among study participants (*n* = 28) *.

Study Measure	Min	Max	Mean	Median	Standard Deviation	*p*-Value
Fagerstrom Test of Nicotine Dependence (10 point scale)						Difference between: 0–6 weeks: *p* < **0.0001**; 0–12 weeks: *p* < **0.0001**
Initial	0	10	4.82	5.0	2.58	
6-Week	0	8	2.75	2.5	2.70	
12-Week	0	8	2.61	2.0	2.71	
PHQ-9 (27 point scale)						Difference between: 0–6 weeks: *p* = 0.71; 0–12 weeks: *p* = 0.91
Initial	0	25	7.68	4.5	7.49	
6-Week	0	24	7.50	5.0	7.16	
12-Week	0	24	7.89	5.0	8.05	
Motivation to stop smoking (10 point scale)						Difference between: 0–6 weeks: *p* = 0.40; 0–12 weeks: *p* = 0.69
Initial	5	10	8.75	9	1.29	
6-Week	5	10	9.0	10	1.36	
12-Week	5	10	8.84	10	1.66	
Confidence to stop smoking (10 point scale)						Difference between: 0–6 weeks: *p* = 0.31; 0–12 weeks: *p* = 0.17
Initial	4	10	7.64	8	1.77	
6-Week	2	10	7.89	8	2.01	
12-Week	2	10	8.29	9	2.05	
Contemplation Ladder (10 point scale)						Difference between: 0–6 weeks: *p* = **0.04**; 0–12 weeks: *p* = 0.07
Initial	5	8	7.11	7	0.88	
6-Week	5	9	7.61	8	1.07	
12-Week	5	10	7.68	8	1.67	

Significant values are bolded. * 26 women completed follow-up; however the two women lost to follow-up are included in the analysis using an intent-to-treat analysis.

**Table 3:** Nicotine use at baseline, 6, and 12 weeks

Time of Measure	Min	Max	Mean	Median	STD	*p*-Value *
Cigarettes smoked per day (*n* = 28) **						
Baseline	3	40	16.39	18.5	7.74	
6-week follow-up	0	20	7.11	6	5.69	Baseline—6 week difference: *p* < 0.0001
12-week follow-up	0	20	5.96	5.5	5.46	Baseline—12 week difference: *p* < 0.0001
E-cigarette cartridges used (*n* = 26, all women remaining in the study at 6 weeks)						
0–6 weeks	<1	84	27.39	21.0	23.65	6–12 week difference: *p* < 0.0001
6–12 weeks:	0	42	8.45	5.25	10.45	
E-cigarette cartridges used (*n* = 16 subjects still using ECs at the 6-week follow-up)						
0–6 weeks	6.5	84	30.06	21.0	22.9	6–12 week difference: *p* < 0.0001
6–12 weeks:	1.0	42	14.79	12.5	9.8	
Seven day point prevalence for abstinence					Number of women (*n* = 28) **	
At 6-weeks					4 (14.3%)	
At 12-weeks					8 (28.6%)	
Number of women who were abstinent at 6-weeks and who remained abstinent at 12-weeks					4 (100%)	
Number of women who continued to use the EC at the 12-week follow-up					16/28 (57.1%)	
Number of women who quit nicotine products entirely (neither smoking cigarettes nor using the EC) at the 12-week follow-up					4/28 (14.3%)	

* *p*-values are based on results of the Wilcoxon signed-rank test. Significant values are in bold. ** 26 women completed follow-up; however the two women lost to follow-up are included in the smoking cessation/reduction analysis using an intent-to-treat analysis.
